# The Monitoring Efficacy of Neurogenic Bowel Dysfunction Treatment on Response (MENTOR) in a Non-Hospital Setting

**DOI:** 10.3390/jcm10020263

**Published:** 2021-01-12

**Authors:** Sofie Dagmar Studsgaard Slot, Simon Mark Dahl Baunwall, Anton Emmanuel, Peter Christensen, Klaus Krogh

**Affiliations:** 1 Department of Hepatology and Gastroenterology, Aarhus University Hospital of Aarhus, DK8200 Aarhus N, Denmark; sofie.studsgaard@hotmail.com (S.D.S.S.); SIMJOR@rm.dk (S.M.D.B.); 2 GI Physiology Unit, University College Hospital, London NW1 2BU, UK; anton.emmanuel@nhs.net; 3 Department of Surgery, Aarhus University Hospital of Aarhus, DK8200 Aarhus N, Denmark; petchris@rm.dk

**Keywords:** SCI, MENTOR, NBD, constipation, fecal incontinence

## Abstract

Background: Most patients with a spinal cord injury (SCI) suffer from neurogenic bowel dysfunction (NBD). In spite of well-established treatment algorithms, NBD is often insufficiently managed. The Monitoring Efficacy of Neurogenic bowel dysfunction Treatment On Response (MENTOR) has been validated in a hospital setting as a tool to support clinical decision making in individual patients. The objective of the present study was to describe clinical decisions recommended by the MENTOR (either “monitor”, “discuss” or “act”) and the use of the tool to monitor NBD in a non-hospital setting. Methods: A questionnaire describing background data, the MENTOR, ability to work and participation in various social activities was sent by mail to all members of The Danish Paraplegic Association. Results: Among 1316 members, 716 (54%) responded, 429 men (61%) and 278 women (39%), aged 18 to 92 (median 61) years. Based on MENTOR, the recommended clinical decision is to monitor treatment of NBD in 281 (44%), discuss change in treatment in 175 (27%) and act/change treatment in 181 (28%). A recommendation to discuss or change treatment was associated with increasing age of the respondent (*p* = 0.016) and with impaired ability to work or participate in social activities (*p* < 0.0001). Conclusion: A surprisingly high proportion of persons with SCI have an unmet need for improved bowel care. The MENTOR holds promise as a tool for evaluation of treatment of NBD in a non-hospital setting.

## 1. Introduction

The term neurogenic bowel dysfunction (NBD) covers gastrointestinal symptoms that complicate lesions or diseases in the central nervous system. NBD is normal among patients with spinal cord injury (SCI), multiple sclerosis, spina bifida or cauda equina syndrome. The most common symptoms are constipation and/or faecal incontinence, which affect more than 80% of SCI patients [1,2]. Symptoms of NBD restrict social activities and impair quality of life [1,2]. Especially, the loss of independence controlling or achieving defecation is burdensome [3,4]. Despite the consequences of NBD and existing stepwise treatment approaches, the management of NBD is usually not systematically evaluated. This may delay initiation of appropriate treatment [5,6,7,8].

Evaluation of NBD is usually based on patient reported symptoms. Several scores exist for assessment of either constipation or faecal incontinence. Unfortunately, most have not been validated for use in patients with neurological disorders and they do not cover the full spectrum of bowel symptoms experienced by such patients. The NBD score is a 10-item score developed and validated among persons with SCI [9]. It has been translated into more than 15 languages and remains the most cited score for description of NBD or as endpoint in clinical trials [9,10,11]. The NBD score correlates with the impact of NBD on the quality of life in persons with NBD, but it was not developed for clinical decision making in individual patients [12].

Monitoring Efficacy of Neurogenic bowel dysfunction Treatment On Response (MENTOR) is a tool to monitor treatment and determine progression of treatment for NBD. It combines three domains: the NBD score, special attention symptoms indicating insufficient treatment, and patient satisfaction with their bowel function. Thus, it offers a holistic outcome that has been shown to be both easy and reliable to use in clinical practice [5,13]. The MENTOR was developed in a hospital setting, and it has been validated among persons with SCI in four European countries and the USA [13]. At present, MENTOR has not been applied in a broader community-based group of people with NBD. Such data is warranted as it will provide valuable information about the need for improved treatment of people with SCI in general and inform whether systematic monitoring of the patient group is required. Most changes in treatment for NBD are decided at scheduled control visits at specialist clinics. If useful in a non-hospital setting, the MENTOR could prove valuable as a tool for patients and caregivers outside specialist clinics to identify who is in need for enhanced treatment of NBD and therefore should be referred to specialist centres.

The aim of the present study was to describe clinical decisions recommended by the MENTOR (either “monitor”, “discuss” or “act”) and the use of the tool to monitor NBD in a non-hospital setting.

## 2. Methods

In this cross-sectional survey, a questionnaire was sent by mail to all 1316 active members of the Danish Paraplegic Association. The Danish Paraplegic Associations is a patient organisation covering more than 35% of Danish persons with SCI from all regions of the country.

All members were mailed the questionnaire at the same time with instructions on how to return the responses by mail. Members who did not respond within 4 weeks were mailed a reminder with the questionnaire. Once the questionnaires were returned, all data were entered twice to minimise transcription errors.

The questionnaire included 29 items describing age; gender; time since spinal cord lesion; function of hand and legs; cutaneous sensibility; previous abdominal surgery; stoma; constipation; method of defecation; bowel habits; faecal incontinence; contact with healthcare providers; satisfaction with current bowel function; and impact of NBD on social activities, ability to work or quality of life. Included in the questionnaire were the NBD score and the MENTOR. Based on the respondent’s description of motor and sensory function, the level of the SCI was described as either cervical or thoracic/lumbar and either sensory and motor complete or incomplete.

Special attention symptoms are symptoms that indicate insufficient management of NBD. Those symptoms were included in the questionnaire and have been described in detail previously [13].

According to MENTOR, all participants were grouped as either green, yellow or red. These groups indicate that symptoms should be monitored (green), a need for discussion of change in treatment (yellow) or a need to change treatment modality for NBD.

According to Danish legislation, questionnaire studies do not need approval from Ethics Committee.

## 3. Statistical Analysis

Statistical analysis was performed in GraphPad Software (Prism 8 8.4.3, GraphPad Software, Inc., San Diego, CA, USA). Results are given as median with range or proportions with confidence interval. For continuous normal data, we used Kruskal Wallis test across the three MENTOR groups, and for categorical data we used chi square test. In the grouping of MENTOR, we considered incomplete responses as no responses to limit potential reporting bias and provide the most conservative estimates. In specific analyses on each item separately, incomplete answers were omitted from the analysis. A *p*-value of less than 0.05 was considered statistically significant.

Among 1316 members of The Danish Paraplegic Association, 716 (54%) responded, 429 men (61%) and 278 women (39%), aged 18 to 92 (median 61) years. Time since the lesion was 2 to 90 (median 20) years. The level of lesion was cervical in 312 (47%) and thoracic or lumbar in 352 (53%). The lesion was sensory complete in 285 (41%) and motor complete in 356 (51%). A total of 79 respondents (11%) had a stoma and were excluded from the following analysis leaving a total 630 respondents. The respondent’s contact to the healthcare system and the follow-up regarding bowel care are summarised in Table 1. In total, 366 (62%) had been seen for follow-up at specialist SCI centres within the last two years and 312 (52%) had discussed bowel care with a healthcare provider. However, 182 (30%) had not discussed methods for bowel care within the last five years.

### 3.1. Neurogenic Bowel Dysfunction Score

Responses to each of the 10 items in the NBD score are shown in Table 2. Median NBD score was 8 (range 0–34). Among respondents, 235 (38%) had no or very minor, 122 (20%) had minor, 141 (23%) had moderate and 123 (20%) had severe NBD.

### 3.2. Satisfaction with Bowel Function

In total, 132 (21%) rated satisfaction with their bowel function within the past 4 weeks as good, 324 (53%) as acceptable, 136 (22%) as bad and 25 (4%) as very bad (Table 3).

### 3.3. Special Attention Symptoms

Special attention symptoms were experienced by 224 (38%). These included intense pain in the abdomen or rectum (*n* = 116, 20%), new or increased bleeding from the anus (*n* = 92, 16%), hospitalisation due to bowel problems within the last year (*n* = 29, 5%), reduction in independence with regard to bowel care (*n* = 51, 9%) and episodes of autonomic dysreflexia related to bowel management (*n* = 87, 15%).

### 3.4. The MENTOR Tool

According to the MENTOR tool, the proposed clinical decision was to “monitor/control” (green) in 281 (44%), “discuss treatment options” (yellow) in 175 (27%) and “act/change treatment” in 181 (28%) (Table 3). Table 3 presents the MENTOR classification before adjusting for special attention symptoms. In total, 134 (21%) changed MENTOR group due to special attention symptoms, and across the MENTOR groups the median (IQR) number of special attention symptoms was 0 (0–0) for green, 0 (0–1) for yellow and 1 (1–2) red.

There was a significant association between the increasing need for change in treatment and age of the respondents (*p* = 0.016). There was no association between response to the MENTOR and time since SCI (*p* = 0.155), gender (*p* = 0.106), sensory completeness (*p* = 0.868) or motor completeness of the lesion (*p* = 0.263).

### 3.5. Effects of Neurogenic Bowel Dysfunction on Daily Life

**Figure 1:**
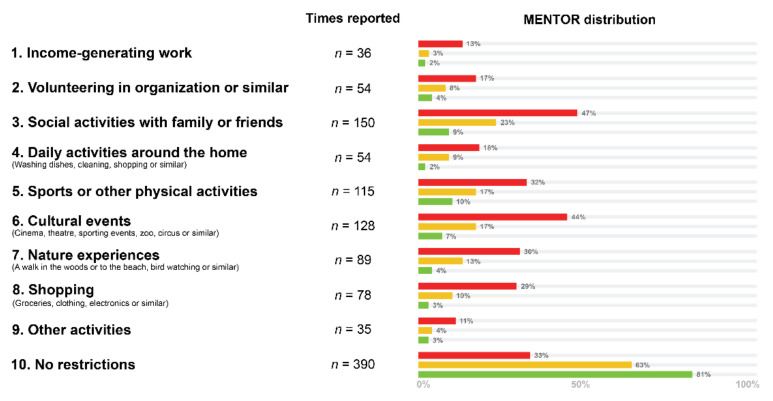
Daily restrictions experienced by the respondents and relative frequency according Monitoring Efficacy of Neurogenic bowel dysfunction Treatment on Response (MENTOR) group. Label: mentor frequencies are derived from the relative count divided by the total number of patients in each of the three MENTOR groups.

Among respondents, 240 (38%) reported that NBD restricted various aspects of daily life (Figure 1). Thus, 36 (6%) reported that NBD prevented them from having income-generating work, 54 (9%) from volunteering in organizations or similar, 150 (24%) from social activities with family or friends, 54 (9%) from daily activities in or around the home (washing dishes, cleaning, shopping or similar), 115 (18%) from sports or other physical activity, 128 (20%) from cultural events (cinema, theatre, concerts, sporting events, zoo, circus or similar), 89 (14%) from nature experiences (a walk in the woods or to the beach, bird watching, star gazing or similar), 78 (12%) from shopping (groceries, clothing, electronics or similar) and 35 (6%) from other activities.

The recommendation from the MENTOR was associated with self-reported impairment of one or more aspects of daily life due to NBD (*p* < 0.0001) (Table 4). Among respondents reporting that NBD caused some restriction of daily life, the MENTOR would recommend “discuss treatment” (yellow) or “act/change treatment” (red) in 77% and “monitor/control” (green) in 23%. If respondents reported no restriction in daily activities, the MENTOR recommended “monitor/control” (green) or “discuss treatment” (yellow) in 85% and “act/change treatment” (red) in 15%. Among the respondents for whom the MENTOR recommended “monitor/control” (green), 81% reported no impairment of daily life because of NBD. Among those for whom MENTOR recommended “act/change treatment” (red), 67% reported some impairment in daily life due to NBD.

## 4. Discussion

The MENTOR was developed as an easy-to-use instrument for assessment of the need for change in bowel care in individuals with SCI [13]. It incorporates the commonly used 10-item NBD score; patient satisfaction with current treatment; and so called “special attention symptoms”, which indicate unsatisfactory bowel management. Based on the MENTOR, the potential need for change in bowel care is classified as either “monitor”, indicating that bowel care is sufficient; “discuss”, indicating that there may be a need for change; and “act”, indicating that bowel care is unsatisfactory and that there is a need for change. The grouping of responses into the three categories corresponds well with the opinion of experts in NBD [13]. The main finding of the present study was that 56% of non-hospitalised persons with SCI had a need for discussion or change of bowel management. This includes 28% who most likely had a serious need for change. The secondary finding was that results from the MENTOR were associated with restriction in various social activities caused by NBD. This further validates the MENTOR as a clinical tool and supports its future use in a non-hospital setting.

Diseases or lesions within the spinal cord disrupt normal bowel function. Anorectal sensation is reduced or lost, rectal evacuation at defecation is reduced and transit time through the colon is prolonged [14,15,16]. The resulting symptom complex is usually termed NBD. Most common symptoms of NBD are constipation and faecal incontinence [2,3]. Neurogenic bowel dysfunction severely restricts social activities and has a negative impact on quality of life. Within recent decades, several new treatment modalities have been introduced against NBD [17,18]. A detailed description of treatment algorithms for NBD is beyond the scope of the present paper. However, a stepwise treatment algorithm has been endorsed and described in detail in previous publications [5,19]. Unfortunately, the improvement in treatment options has not yet sufficiently changed clinical practice. Thus, most persons with NBD due to SCI have used the same method for bowel care in spite of 40% being dissatisfied with their bowel function. Insufficiently treated NBD is unfortunate because correct treatment is both cost-effective and improves the quality of life of the patient [20,21,22]. It is for this reason that the MENTOR instrument was developed, to identify individuals at need for change of method for bowel care.

Neurogenic bowel dysfunction is a clinical diagnosis. Thus, several symptom-based instruments for assessment of NBD have been developed and recently critically reviewed [17]. The most commonly used and best validated tool was found to be the NBD score, which includes 10 items describing various aspects of NBD [9]. Each item is weighted from its impact on quality of life. The score was developed among Danish persons with SCI, and it was later validated among patients with multiple sclerosis. Lately, it has been incorporated in the International SCI Bowel Function Data Set [19]. The NBD score was not created for decision making in individual patients. For this purpose, and to facilitate the progression through treatment, the MENTOR was developed.

The MENTOR includes three dimensions: the NBD score, patient satisfaction with current treatment of NBD and special attention symptoms. The latter are single symptoms or experiences that strongly indicate severe bowel dysfunction whether related to NBD or not. Interestingly, 38% of respondents reported one or more of such symptoms, the commonest being pain or bleeding from the rectum. These symptoms do not only indicate that treatment of NBD is insufficient, but they may also be alarm symptoms warning the clinician that other pathology could be present. Spinal cord injury mainly affects the colorectum and the anal canal. The effects of NBD are, however, not limited to these segments. Fynne et al. found that transit through the upper gastrointestinal tract was delayed in persons with SCI [23]. Moreover, constipation or anorectal digitation during bowel care may cause autonomic dysreflexia with very high blood pressure in persons with SCI above the sixth thoracic level [6,24]. Insufficient treatment of NBD increases the risk of urinary tract infections and causes hospitalization [18,20,25]. Hence, some of the special attention symptoms were included to cover consequences of NBD beyond bowel symptoms.

The awareness about NBD has increased dramatically in recent decades [3]. It is increasingly recognised that autonomic consequences of SCI should be considered equally with the impairment of motor function [17,19,26]. Most persons with SCI rate NBD among the three most bothersome consequences of SCI. Even though NBD is life-long, it is not a stable condition. Constipation and impairment of quality of life become more severe with time since injury [27,28,29]. This calls for life-long control of bowel function. We find that MENTOR qualifies for this purpose both among patients seen in hospital and in the community.

There are limitations to the present study. To ensure an acceptable response rate to our survey, we had to keep the mailed questionnaire short and simple. For this reason, we choose to compare the recommendations from the MENTOR with the self-reported impact on various aspects of daily life. These items were developed by members of the Danish Paraplegic Association but have not been validated. The inclusion of a validated score for quality of life would have been preferable. In the previous study on the MENTOR tool, the recommendations “monitor/control” (green) and “act/change treatment” (red) correlated well with the opinion of experienced experts. In the present study, there was a fair correlation between the same recommendations from the MENTOR and the self-reported impairment of daily life. Like in a previous publication, the middle group “discuss” (yellow) performed less well [13]. In our opinion this does not disqualify the MENTOR, because a recommendation of “discuss” will lead to a decision of monitoring or to act after the discussion with the patient. The majority of respondents (61%) were males. We do not know the exact male/female proportion among members of the Danish Paraplegic association, but there are significantly more male than female members. Hence, the gender distribution among respondents most likely reflects that of the association.

The present study was restricted to adult persons with NBD due to SCI. Several other groups of patients suffer from NBD too. Thus, NBD is reported by approximately 50% of patients with multiple sclerosis or spina bifida. The NBD score has proven useful in patients with NBD caused by multiple sclerosis [8,30]. Future studies will determine whether the MENTOR is applicable outside an SCI population. Healthcare systems are changing around the world, and electronic collection and remote monitoring of patients reported outcomes will without doubt become a part of clinical monitoring of future patients. The MENTOR is easily understandable and takes approximate 5 min to complete [13]. In the present study, we found it useful as part of a survey.

## 5. Conclusions

In conclusion, we found that 28% of non-hospitalised persons with SCI had bowel symptoms mandating a change in methods for bowel care and another 27% had a need for discussion of a potential change in treatment strategy. Moreover, recommendations from the MENTOR correlated with self-reported impairment of daily activities caused by NBD.

## Figures and Tables

**Table 1:** The respondent’s contact to the healthcare system.

	When Have You Last Seen a Doctor/Nurse Because of SCI?	When Have You Last Discussed Your Bowel Function with a Doctor/Nurse?
Less than one year ago	166 (28%)	156 (26%)
1–2 years	200 (34%)	156 (26%)
>2–5 years	147 (25%)	113 (19%)
More than 5 years	63 (11%)	78 (13%)
Never	20 (3%)	105 (17%)
Missing values	34 (5%)	22 (3%)

SCI: Spinal cord injury.

**Table 2:** The response to the 10 items of the Neurogenic Bowel Dysfunction (NBD).

NBD Score	*n* (%)
**1. How often do you defacate?**	
Daily	335 (53.3%)
2–6 times per week	281 (44.7%)
Less than once per week	12 (1.9%)
**2. How much times do you spend on each defaecation?**	
Less than 30 min.	402 (64.1%)
31–60 min.	190 (30.3%)
More than an hour	35 (5.6%)
**3. Do you experience uneasiness, sweating or headaches during or after defaecation?**	
Yes	150 (23.9%)
No	478 (76.1%)
**4. Do you take medication (tablets) to treat constipation?**	
Yes	281 (45.0%)
No	344 (55.0%)
**5. Do you take medication (drops or liquid) to treat constipation?**	
Yes	170 (27.2%)
No	455 (72.8%)
**6. How often do you use digital evacuation?**	
Less than once per week (score 0)	329 (52.6%)
Once or more per week (score 6)	297 (47.4%)
**7. How often do you have involuntary defaecation?**	
Daily	5 (0.8%)
1–6 times a week	19 (3.0%)
3–4 times a month	83 (13.3%)
A few times a year or less	518 (82.9%)
**8. Do you take medication to treat faecal incontinence?**	
Yes	23 (3.7%)
No	605 (96.3%)
**9. Do you experience uncontrollable flatus?**	
Yes	379 (60.4%)
No	248 (39.6%)
**10. Do you have peri-anal skin problems?**	
Yes	118 (19.0%)
No	508 (81.2%)

**Table 3:** Distribution of responses according to the NBD score and patient satisfaction before adjusting for special attention symptoms.

NBD Score	Patient Satisfaction			
	Good	Acceptable	Poor	Very Poor
14 or more	9 (1.5%)	44 (7.1%)	51 (8.3%)	17 (2.8%)
10–13	27 (4.4%)	75 (12.2%)	36 (5.8%)	3 (0.5%)
0–9	96 (15.6%)	205 (33.3%)	49 (7.9%)	5 (0.8%)

Based on 617 respondents, 20 (3%) respondents had incomplete responses to calculate the NDB score or did not answer patient satisfaction. Percentages are of the total number of complete responses.

**Table 4:** Self-reported restriction in various aspects of daily life within the three MENTOR groups.

	Green *n* = 281	Yellow *n* = 175	Red *n* = 181
Income-generating work	6 (2.14%)	6 (3.43%)	24 (13.26%)
Volunteering in organization or similar	10 (3.56%)	14 (8%)	30 (16.57%)
Social activities with family or friends	24 (8.54%)	41 (23.43%)	85 (46.96%)
Daily activities around the home (washing dishes, cleaning, shopping or similar)	5 (1.78%)	16 (9.14%)	33 (18.23%)
Sports or other physical activities	28 (9.96%)	29 (16.57%)	58 (32.04%)
Cultural events (cinema, theatre, sporting events, zoo, circus or similar)	19 (6.76%)	29 (16.57%)	80 (44.2%)
Nature experiences (a walk in the woods or to the beach, bird watching or similar)	12 (4.27%)	22 (12.57%)	55 (30.39%)
Shopping (groceries, clothing, electronics or similar)	8 (2.85%)	18 (10.29%)	52 (28.73%)
Other activities	9 (3.2%)	7 (4%)	19 (10.5%)
No restrictions	228 (81.14%)	110 (62.86%)	59 (32.6%)
